# Práticas profissionais e compromissos no cuidado às pessoas que vivem com HIV/aids

**DOI:** 10.1590/0102-311XPT156225

**Published:** 2026-07-24

**Authors:** Fernanda Karla Metelski, Betina Hörner Schlindwein Meirelles, Marcelle Miranda da Silva, Alacoque Lorenzini Erdmann, Carine Vendruscolo, Wilson Jorge Correia Pinto de Abreu

**Affiliations:** 1 Programa de Pós-graduação em Enfermagem, Universidade do Estado de Santa Catarina, Chapecó, Brasil.; 2 Programa de Pós-graduação em Enfermagem, Universidade Federal de Santa Catarina, Florianópolis, Brasil.; 3 Programa de Pós-graduação em Enfermagem, Universidade Federal do Rio de Janeiro, Rio de Janeiro, Brasil.; 4 Escola Superior de Enfermagem do Porto, Universidade do Porto, Porto, Portugal.

**Keywords:** HIV, Atenção Primária à Saúde, Gestão em Saúde, Estigma Social, Teoria Fundamentada, HIV, Primary Health Care, Health Management, Social Stigma, Grounded Theory, VIH, Atención Primaria de Salud, Gestión en Salud, Estigma Social, Teoría Fundamentada

## Abstract

Este estudo objetivou compreender as práticas profissionais no cuidado às pessoas que vivem com HIV/aids frente aos compromissos globais estabelecidos para o fim da epidemia de aids até 2030, em um município prioritário de Santa Catarina, Brasil. Trata-se de uma pesquisa qualitativa ancorada na vertente construtivista da Teoria Fundamentada nos Dados, realizada nos cenários da atenção primária à saúde e da atenção especializada, valendo-se de entrevistas em profundidade com 45 participantes, dentre profissionais da assistência, gestores e pessoas que vivem com HIV/aids, realizadas entre 2020 e 2021. A análise dos dados originou a categoria central “Desvelando práticas profissionais locais voltadas aos compromissos globais para o enfrentamento da epidemia de HIV/aids”, sustentada por sete subcategorias que revelam a ampliação da testagem rápida oportuna com acolhimento e aconselhamento, garantia de acesso, diagnóstico precoce e início imediato da terapia antirretroviral, o engajamento ao regime terapêutico e suas contradições, a estigmatização social, o medo e a superação frente ao HIV/aids, e as limitações dos sistemas de informação. Sobressaíram as práticas relacionadas a aspectos preventivos e clínicos com protagonismo do enfermeiro. Observou-se uma abordagem incipiente quanto ao enfrentamento da estigmatização social vivenciada pelas pessoas com HIV/aids em diferentes dimensões: por elas próprias, por seus familiares, por profissionais de saúde e no convívio social. Superar a epidemia de HIV/aids demanda estratégias articuladas e equitativas que considerem os determinantes sociais da saúde, ampliem o acesso e promovam o bem-estar das pessoas que vivem com HIV/aids.

## Abstract (en)

This study sought to understand the professional practices in caring for people living with HIV/AIDS considering the global commitments to end the AIDS epidemic by 2030 in a priority municipality of Santa Catarina, Brazil. Qualitative research based on the constructivist branch of grounded theory was carried out in primary health and specialized care with in-depth interviews with 45 participants, including healthcare providers, managers, and people living with HIV/AIDS, from 2020 to 2021. Data analysis obtained the core category “Unveiling local professional practices aimed at global commitments to confront the HIV/AIDS epidemic”, supported by seven subcategories that evince the expansion of timely rapid testing with reception and counseling, guarantee of access, early diagnosis and immediate start of antiretroviral therapy, engagement with the therapeutic regimen and its contradictions, social stigmatization, fear and overcoming HIV/AIDS, and the limitations of information systems. Practices related to preventive and clinical aspects stood out, with nurses playing a leading role in them. Coping with the social stigmatization by people living with HIV/AIDS experience still features an incipient approach in several dimensions: themselves, their families, healthcare providers, and social life. Overcoming the HIV/AIDS epidemic requires articulated and equitable strategies that consider the social determinants of health, expand access, and promote the well-being of people living with HIV/AIDS.

## Resumen

Este estudio tuvo como objetivo comprender las prácticas profesionales en el cuidado de las personas que viven con VIH/sida a la luz de los compromisos mundiales establecidos para poner fin a la epidemia del sida para el 2030, en un municipio prioritario de Santa Catarina, Brasil. Se trata de una investigación cualitativa basada en el enfoque constructivista de la Teoría Fundamentada en Datos, realizada en entornos de atención primaria de salud y especializada, con base en entrevistas en profundidad con 45 participantes, entre los que se incluyen profesionales de la asistencia, gestores y personas que viven con VIH/sida, llevadas a cabo entre el 2020 y el 2021. El análisis de datos dio como resultado la categoría central “Revelación de prácticas profesionales locales orientadas a los compromisos mundiales para afrontar la epidemia del VIH/sida”, respaldada por siete subcategorías que revelan la ampliación de las pruebas rápidas oportunas con apoyo y asesoramiento, el acceso garantizado, el diagnóstico precoz y el inicio inmediato de la terapia antirretroviral, la participación en el régimen terapéutico y sus contradicciones, la estigmatización social, el miedo y la superación de los desafíos frente al VIH/sida, y las limitaciones de los sistemas de información. Sobresalieron las prácticas relacionadas con los aspectos preventivos y clínicos, en las que los enfermeros desempeñaron un papel protagonista. Se observó un enfoque incipiente con respecto al afrontamiento de la estigmatización social que experimentan las personas con VIH/sida en diferentes dimensiones: por parte de ellas mismas, de sus familias, de los profesionales de la salud y en la vida social. Para superar la epidemia del VIH/sida se necesitan estrategias coordinadas y equitativas que tengan en cuenta los determinantes sociales de la salud, amplíen el acceso y promuevan el bienestar de las personas que viven con VIH/sida.

## Introdução

A Agenda 2030 para o Desenvolvimento Sustentável foi adotada pelos Estados-Membros da Organização das Nações Unidas (ONU), incluindo o Brasil. Os Objetivos do Desenvolvimento Sustentável (ODS) propõem metas para garantir que todas as pessoas possam usufruir da paz e da prosperidade em todos os lugares, oportunizando a criação de cultura de bem-estar e saúde planetária, o fortalecimento da prevenção de doenças, a proteção e a promoção da saúde ^1^. 

“Saúde e bem-estar”, terceiro ODS, busca assegurar uma vida saudável e promover o bem-estar para todas as pessoas em qualquer idade, incluindo a eliminação da aids como problema de saúde pública até 2030. Para reverter a tendência de propagação do HIV e atingir a meta 95-95-95, propõe-se diagnosticar 95% das pessoas vivendo com HIV/aids, 95% das pessoas diagnosticadas estar em tratamento antirretroviral (ARV) e 95% das pessoas em tratamento com carga viral indetectável ^2^ ^,^ ^3^. Em 2023, o Brasil atingiu 91-83-95, sinalizando a importância das parcerias estratégicas com a sociedade civil, científica e internacional para o fim da epidemia ^4^.

Ademais, a “educação de qualidade”, quarto ODS, propõe educação para o desenvolvimento sustentável e estilos de vida sustentáveis e direitos humanos ^5^, e está alinhada à necessidade de ação conjunta e educação em saúde. No Brasil, o enfrentamento da epidemia está historicamente articulado à Política Nacional de HIV/aids, uma das primeiras políticas públicas de saúde a incorporar ações intersetoriais e de direitos humanos como eixos estruturantes. A política orienta a prevenção, o diagnóstico, o tratamento e a promoção da saúde das pessoas que vivem com HIV/aids, com base na universalidade, integralidade e equidade do SUS ^6^. 

Desde o aparecimento dos primeiros casos de aids no final do século XX, 88,4 milhões de pessoas foram infectadas pelo HIV em todo o mundo, e 42,3 milhões morreram de doenças relacionadas à aids. Em 2023, 39,9 milhões de pessoas viviam com HIV e 30,7 milhões de pessoas (76,9%) tiveram acesso a ARV ^3^. As maiores quedas na incidência e na mortalidade por HIV foram alcançadas na África Subsaariana, enquanto taxas crescentes são registradas em regiões como Europa Central e Oriental, Ásia Central, Norte da África e Oriente Médio ^7^. No Brasil, o Estado de Santa Catarina é considerado prioritário para o enfrentamento da epidemia de aids. Em 2023, Santa Catarina apresentou uma taxa de detecção de 34 casos de HIV por 100 mil habitantes em adultos, sendo superior à taxa nacional de 21,8. Em relação a aids, a taxa de detecção foi de 17,1 em Santa Catarina e de 17,8 no Brasil, com coeficiente de mortalidade de 5,5 por 100 mil habitantes em Santa Catarina, superior ao coeficiente nacional de 4,8 ^3^.

As diferentes estratégias lançadas por organizações internacionais e os avanços alcançados ao longo dos anos não se distribuíram de forma uniforme entre os países no enfrentamento à epidemia de HIV e na redução da mortalidade associada à aids. Na região das Américas, a diminuição da mortalidade foi inferior ao esperado. A morbimortalidade por aids está diretamente relacionada à imunodeficiência das pessoas, resultante do não engajamento ao tratamento, do abandono da terapia ARV, do diagnóstico tardio e da evolução para doença avançada ^2^ ^,^ ^8^.

As metas para erradicar a epidemia de HIV são ambiciosas, viáveis e têm potencial para reduzir a mortalidade global relacionada à infecção ^9^. A implementação de estratégias baseadas em evidências, centradas nas pessoas vivendo com HIV/aids e nas comunidades, pode ampliar o alcance e a efetividade das ações preventivas, especialmente quando conduzidas com liderança eficaz e compassiva ^10^. O enfrentamento da epidemia exige ações coordenadas em níveis local, nacional e global, considerando o contexto epidemiológico, sociocultural e comportamental, bem como fatores como estigma, criminalização e a necessidade de serviços de saúde complementares. Além de dados confiáveis, são necessárias pesquisas contínuas para aprimorar e implementar intervenções preventivas de forma mais eficaz ^9^.

Os testes rápidos de HIV têm sido realizados no Brasil desde 2011, inclusive no âmbito da atenção primária. Dentre as estratégias adotadas para a eliminação da aids e da transmissão do HIV está a criação de linhas de cuidado regionalizadas, com foco na integralidade do cuidado e articulação das ações entre atenção primária à saúde (APS), atenção especializada e vigilância em saúde, respeitando as realidades e pactuações locais ^4^. 

A APS favorece o diagnóstico precoce e o acompanhamento de pessoas soropositivas assintomáticas, já a atenção especializada atua nos casos sintomáticos. A integralidade do cuidado exige articulação entre os serviços, embora haja variações nos modelos adotados pelos municípios ^11^, o que requer compreender os avanços e desafios no combate à epidemia de HIV/aids no contexto local. A descentralização amplia o acesso, mas impõe desafios como a gestão do cuidado e a garantia do sigilo ^12^. Este trabalho adota o referencial da gestão do cuidado, orientado pelas necessidades singulares dos indivíduos em suas múltiplas dimensões: individual, familiar, profissional, organizacional, sistêmica e societária ^13^.

Assim, delineou-se como questão de pesquisa: De que maneira as práticas adotadas por profissionais da assistência e gestores no cuidado às pessoas que vivem com HIV/aids em um município prioritário respondem aos compromissos globais para o enfrentamento da epidemia de HIV/aids até 2030? O objetivo do trabalho é compreender as práticas profissionais no cuidado às pessoas que vivem com HIV/aids, frente aos compromissos globais estabelecidos para o fim da epidemia de aids até 2030, em um município prioritário de Santa Catarina.

## Método

Estudo qualitativo que utilizou o referencial metodológico da Teoria Fundamentada nos Dados na vertente Construtivista (TFDC). Nessa vertente da TFD, os dados e as teorias são construídos na interação e no envolvimento com as pessoas, com base nas experiências compartilhadas, nas relações estabelecidas com os participantes, e as suas expressões traduzem construções da realidade e de significados, ou seja, um retrato interpretativo do mundo. O pesquisador não é neutro, possui sua maneira de usar a linguagem, suas opiniões e valores ^14^.

A TFDC tem características estruturantes comuns às demais vertentes que envolvem a coleta e análise de dados simultaneamente, a comparação constante dos dados, a amostragem teórica, o desenvolvimento de memorandos e diagramas que contribuem para a análise reflexiva e comparativa, e um processo analítico que orienta o desenvolvimento teórico. As características específicas da vertente construtivista contemplam a possibilidade do uso da literatura em todas as etapas de análise e de modo compilado ao final, e um sistema de codificação aberto e composto minimamente por uma etapa inicial e uma focalizada ^15^.

O estudo foi realizado na APS por ser o principal acesso aos serviços de saúde, abrangendo 19 Centros de Saúde da Família, e no Serviço de Atenção Especializada (SAE) de um município do oeste de Santa Catarina que conta com uma população estimada em 275 mil habitantes e é prioritário para a erradicação da aids. Rotineiramente, os profissionais da APS realizam aconselhamento e oferecem testes rápidos para HIV, sífilis e hepatites B e C, além de encaminhar os casos com resultado positivo para o SAE.

O SAE é referência para 36 municípios, tem uma unidade dispensadora de medicamentos antirretrovirais e uma equipe multiprofissional composta por uma médica infectologista, três enfermeiros, um auxiliar de Enfermagem, três técnicos de Enfermagem, uma farmacêutica, uma psicóloga e uma assistente social A equipe oferece atendimentos individuais e em grupos para pessoas que vivem com HIV/aids e seus parceiros sorodiferentes, realiza capacitações para as equipes da APS, permanecendo disponível para responder aos questionamentos destes profissionais, ações preventivas junto a empresas e instituições, mutirões de testagem em espaços públicos, e busca ativa das pessoas em atraso ou abandono de tratamento. O serviço também realiza atendimento, acompanhamento e investigação de crianças expostas ao HIV por transmissão vertical, e indica a profilaxia pós-exposição (PEP) em casos de violência sexual, exposição ocupacional e relações sexuais consentidas em que não houve o uso do preservativo.

Em 2022, o número de pessoas vivendo com HIV/aids no município onde o serviço está situado, em acompanhamento no SAE, era de 1.112, sendo 622 do sexo masculino e 490 do feminino, dentre os quais, 19 gestantes e 30 crianças em investigação. O número acumulado era de 2.349 pessoas vivendo com HIV/aids desde 1984 nos 36 municípios da região. Em 2023, a taxa de detecção de aids em Santa Catarina foi de 25,8/100 mil habitantes, ocupando a quarta posição nacional, e no município onde foi realizado o estudo a taxa atingiu 30,6 ^16^. Em Santa Catarina, 12 municípios assinaram a *Declaração de Paris* em 2018, pois o estado é considerado um dos mais afetados pela epidemia de HIV/aids no Brasil.

Os participantes da amostragem inicial, selecionados por conveniência com base nos territórios da APS com maior número de pessoas que vivem com HIV/aids, foram profissionais da assistência e gestores. O convite para participar do estudo, efetuado por telefone, respeitou os seguintes critérios de inclusão: atuar no serviço há pelo menos seis meses e realizar algum tipo de ação ou atendimento relacionado ao HIV/aids ou coordenar/gerenciar o respectivo serviço. Profissionais da assistência e gestores afastados por qualquer motivo durante o período de coleta de dados não participaram do estudo. As pessoas que vivem com HIV/aids foram convidadas a participar pelos profissionais de saúde, sendo contatadas aquelas que se encontravam na fase de aceitação da sua condição de viver com HIV/aids.

Dentre os participantes convidados, 11 não aceitaram participar das entrevistas individuais em profundidade, entre agosto de 2020 e novembro de 2021, realizada por uma enfermeira em doutoramento que atuou na Secretaria de Saúde do município onde foi realizado o trabalho até dois anos antes da coleta de dados.

A principal questão do instrumento semiestruturado que deu origem ao estudo foi: Poderia descrever as práticas que você desenvolve nesse serviço e que considera que promovem a ampliação do diagnóstico, o acesso e engajamento das pessoas que vivem com HIV/aids ao tratamento? Essa pergunta está alinhada à questão central da pesquisa: De que maneira as práticas adotadas por profissionais da assistência e gestores no cuidado às pessoas que vivem com HIV/aids em um município prioritário respondem aos compromissos globais para o enfrentamento da epidemia de HIV/aids até 2030? Para as pessoas que vivem com HIV/aids a pergunta inicial foi: Você poderia me contar sobre como é viver com HIV/aids? Este trabalho está fundamentado na hipótese de que as práticas profissionais locais estão alinhadas aos compromissos globais, mas encontram barreiras estruturais e resistências que interferem no cuidado às pessoas que vivem com HIV/aids.

Não foi realizado teste piloto e as entrevistas foram realizadas remotamente por meio de tecnologias de informação e comunicação ou presencialmente em sala privativa no serviço de saúde, a depender das recomendações de distanciamento social impostas pela pandemia de COVID-19. Estavam presentes apenas o entrevistado e a pesquisadora principal, que teve a oportunidade de relatar o seu interesse pelo tópico da pesquisa e as razões para desenvolvê-la, previamente à leitura do Termo de Consentimento Livre e Esclarecido (TCLE).

Seguindo o método da TFDC, a coleta e a análise foram realizadas concomitantemente até a saturação dos dados, ou seja, quando novas ideias deixaram de surgir entre os dados e a categoria principal foi consolidada ^14^ ^,^ ^17^; este processo foi discutido pelos autores na etapa de avaliação da tese. Nenhuma entrevista precisou ser repetida. Os dados analisados, por dois codificadores, direcionaram a amostragem teórica para a constituição do segundo grupo amostral com profissionais que atuavam no SAE, no qual todos aceitaram participar. Para o terceiro grupo amostral, formado por pessoas que vivem com HIV contatadas pelos enfermeiros gestores do SAE e da APS, e direcionadas para a pesquisadora após o aceite prévio, os critérios de inclusão consistiam em serem maiores de 18 anos, com autonomia plena, residentes no município do estudo, e já engajados no tratamento.

As entrevistas foram gravadas, transcritas e encaminhadas aos participantes para validação, que as retornaram sem alterações; a média de duração das entrevistas foi de uma hora, variando entre vinte minutos e duas horas. Todo material foi organizado e inserido no software Atlas.ti (http://atlasti.com/) que permitiu o armazenamento, gerenciamento e recuperação dos dados de modo a facilitar as comparações. Memorandos e diagramas foram desenvolvidos durante e após as entrevistas para contribuir com o processo de análise ^14^. 

A análise foi desenvolvida nas fases de codificação inicial, focalizada, axial e teórica. A codificação inicial estudou os fragmentos dos dados em busca de compreendê-los com base nos significados e experiências dos participantes. Na codificação focalizada esses dados foram agrupados, classificados, sintetizados e organizados em eixos de análise ou categorias e subcategorias. A codificação axial permitiu organizar condições e situações que determinam a estrutura do fenômeno. A codificação teórica entrelaçou e integrou os códigos, ou seja, a história que foi fragmentada, orientando-a analiticamente ^14^. 

A pesquisa respeitou todos os preceitos éticos e foi aprovada pelo Comitê de Ética em Pesquisa da Universidade Federal de Santa Catarina (parecer consubstanciado nº 3.956.203/2020). A fim de garantir a confidencialidade da identidade, privacidade, proteção da imagem e não estigmatização dos participantes da pesquisa, foram utilizados códigos com letras e números sequenciais por grupo amostral e participantes, sendo: o primeiro grupo amostral composto pelos profissionais da APS, identificados de ProfissionalAPS1 a ProfissionalAPS24; o segundo formado pelos profissionais do SAE, de ProfissionalSAE25 a ProfissionalSAE35; e o terceiro grupo amostral constituído por pessoas que vivem com HIV/aids, de PessoaVHA36 a PessoaVHA45 ^18^.

## Resultados

O trabalho foi composto por 45 pessoas, sendo: 24 profissionais das equipes de saúde da família no primeiro grupo amostral; 11 profissionais do SAE no segundo; e dez pessoas que vivem com HIV no terceiro grupo amostral. 

Para o primeiro e segundo grupos amostrais (Tabela 1), a faixa etária dos profissionais variou entre 25 e 57 anos, em sua maioria enfermeiros(as) (n = 25), mulheres (n = 33), e com escolaridade em nível de pós-graduação ou mais (n = 28). Em relação à função, todos os entrevistados acumulavam as funções de coordenação e assistência.

**Tabela 1:** Caracterização socioeducacional dos participantes do primeiro e segundo grupos amostrais, sendo os profissionais que atuam na atenção primária à saúde (APS) e os profissionais que atuam no Serviço de Atenção Especializada (SAE), respectivamente.

Características	Profissionais da APS (n = 24)		Profissionais do SAE (n = 11)	
	n (%)	Média ± DP	n (%)	Média ± DP
Idade (anos)		37,4 ± 7,7		43,2 ± 8,9
25-29	5 (20,8)		0 (0,0)	
30-39	10 (41,7)		5 (45,5)	
40-49	8 (33,3)		3 (27,3)	
50-57	1 (4,2)		3 (27,3)	
Gênero autodeclarado				
Feminino	23 (95,8)		10 (90,9)	
Masculino	1 (4,2)		1 (9,1)	
Profissão				
Enfermeiro(a)	21 (87,5)		3 (27,3)	
Técnico(a) ou auxiliar de Enfermagem	1 (4,2)		4 (36,4)	
Médica	1 (4,2)		1 (9,1)	
Cirurgiã-dentista	1 (4,2)		0 (0,0)	
Assistente social	0 (0,0)		1 (9,1)	
Farmacêutica	0 (0,0)		1 (9,1)	
Psicóloga	0 (0,0)		1 (9,1)	
Tempo de formação (anos)		13,4 ± 6,6		17 ± 9,0
2-9	7 (29,2)		2 (18,2)	
10-19	12 (50,0)		5 (45,5)	
20-30	5 (20,8)		4 (36,4)	
Escolaridade				
Ensino Médio	1 (4,2)		2 (18,2)	
Ensino Superior	1 (4,2)		3 (27,3)	
Pós-graduação	18 (75,0)		6 (54,6)	
Mestrado acadêmico	4 (16,4)		0 (0,0)	
Função atual				
Assistência	8 (33,3)		9 (81,8)	
Coordenação e assistência	16 (66,7)		1 (9,1)	
Administrativa	0 (0,0)		1 (9,1)	

DP: desvio padrão.

Fonte: elaboração própria.

As pessoas que vivem com HIV (Tabela 2), pertenciam a uma ampla faixa etária, entre 23 e 70 anos, sendo cinco mulheres, dois homens homossexuais, um homem transexual e dois homens. A maioria com escolaridade até o Ensino Médio (n = 7) e o tempo em que vive com HIV entre 1 e 40 anos.

**Tabela 2:** Caracterização socioeducacional dos participantes do terceiro grupo amostral, ou seja, as pessoas que vivem com HIV/aids.

Características	Pessoas que vivem com HIV/aids (n = 10)		
	n (%)	Média ± DP
Idade (anos)		45,4 ± 15,9
23-29	2 (20)	
30-49	5 (50)	
50-69	2 (20)	
70	1 (10)	
Gênero autodeclarado		
Feminino	5 (50)	
Homem homossexual	2 (20)	
Homem transexual	1 (10)	
Masculino	2 (20)	
Estado civil		
Solteiro(a)	4 (40)	
Casado(a)	3 (30)	
Divorciado(a)/Separado(a)	2 (20)	
Viúvo(a)	1 (10)	
Profissão/Ocupação		
Aposentada	1 (10)	
Arquiteto	1 (10)	
Consultora de vendas	1 (10)	
Profissional do sexo	1 (10)	
Pedreiro	1 (10)	
Recebe benefício social	1 (10)	
Representante comercial	1 (10)	
Separadora de mercadorias	1 (10)	
Serviços gerais	1 (10)	
Vendedor	1 (10)	
Escolaridade		
Ensino Fundamental completo	2 (20)	
Ensino Fundamental incompleto	2 (20)	
Ensino Médio	3 (30)	
Ensino Superior completo	2 (20)	
Ensino Superior em curso	1 (10)	
Tempo que vive com HIV (anos)		15,7 ± 11,7
1-5	2 (20)	
6-10	1 (10)	
11-15	2 (20)	
16-20	1 (10)	
21-25	3 (30)	
40	1 (10)	

DP: desvio padrão.

Fonte: elaboração própria.

A análise e integração dos dados revelou a categoria central “Desvelando práticas profissionais locais voltadas aos compromissos globais para o enfrentamento da epidemia de HIV/aids”, sustentada em sete subcategorias: “Realizando os testes rápidos”; “Ampliando o diagnóstico precoce”; “Promovendo o engajamento ao regime terapêutico”; “Entendendo as contradições que envolvem o engajamento ao regime terapêutico”; “Desvelando o tabu, preconceito, discriminação e estigma”; “Vivenciando o medo e a superação profissional frente ao HIV/aids”; e “Utilizando sistemas de informação e alguns indicadores”. 

A testagem e ampliação do diagnóstico está representada na interação entre duas subcategorias. “Realizando os testes rápidos” revela que a APS constitui a principal porta de entrada para o diagnóstico de HIV/aids e os testes estão amplamente disponíveis em toda a rede de atenção à saúde. O aconselhamento, testagem e encaminhamento dos casos positivos para o SAE é realizado principalmente pelo enfermeiro, que é considerado o profissional de referência na APS.

Os testes são oferecidos e realizados durante os atendimentos, por procura espontânea ou indicação de amigos, e em espaços públicos durante os eventos de saúde, ocasião que requer atenção com a privacidade. Apesar do amplo acesso para a testagem rápida na APS, há divergência de opiniões sobre a realização dos testes como rotina porque demanda tempo. Então, alguns profissionais optam por solicitar os exames laboratoriais.

“*E acho que a melhor forma além das campanhas é* [a testagem] *no dia a dia. Então, sempre que eles vêm, é realizado*” (ProfissionalAPS1).

“...*Se eu fizer a coleta do preventivo e mais o teste rápido eu fico uma hora com cada paciente*” (ProfissionalAPS23).

Na testagem, o aconselhamento permite ouvir as pessoas, suas dúvidas, demandas e os motivos para realizar os testes, como a relação sexual desprotegida, desconfiança do parceiro, suspeita de ter adquirido sífilis, trabalhar como profissional do sexo, entre outros.

Durante a pandemia de COVID-19, houve interrupção da realização dos testes devido a orientação de distanciamento social, somado ao receio das pessoas e profissionais contraírem a infecção respiratória. Na retomada da testagem houve baixa procura em alguns serviços, enquanto em outros o número de testes aumentou. 

“...*a questão realmente do diagnóstico ficou prejudicada* [no início da pandemia] *por muito tempo nas unidades de saúde* (...)*. E essas ações para testagem, todas ficaram suspensas*” (ProfissionalSAE2).

Na subcategoria “Ampliando o diagnóstico precoce”, sobressai a solicitação de sorologias nos atendimentos e na rotina do pré-natal envolvendo também o parceiro, o rastreamento de contatos, a testagem para a população imigrante e a abordagem do tema infecções sexualmente transmissíveis (IST) nos grupos com usuários, nas reuniões de Conselhos Locais de Saúde e no Programa Saúde na Escola. 

“*Em todos os grupos que nós temos na Unidade, nós abordamos esse assunto.* (...) *antes de nós começarmos esses grupos a gente faz uma bateria de exames em todos os participantes, inclusive os testes de IST/HIV*” (ProfissionalAPS20).

O engajamento ao regime terapêutico e suas contradições estão representados na interação entre duas subcategorias. “Promovendo o engajamento ao regime terapêutico” desvela uma questão individual multifatorial que implica questões familiares, vínculo entre a pessoa que vive com HIV e os profissionais, compreensão sobre o HIV e como seguir o regime terapêutico adequadamente, questões psicológicas, sociais, sexuais, negação, crenças, medo e preconceito. 

Aceitar a condição de soropositividade, sentir-se comprometido com a própria saúde e estabelecer vínculo de confiança com o profissional são premissas para o engajamento. Os fatores que promovem o engajamento são a busca ativa, o envolvimento do parceiro, encaminhamento com garantia de atendimento, responder dúvidas, ajustar ou substituir os medicamentos quando necessário, trocar informações entre profissionais de saúde, fornecer leite no SAE para crianças de mães soropositivas e oferecer suporte para os familiares apoiarem as pessoas que vivem com HIV/aids.

“*Muitas vezes são problemas sociais, familiares que dificultam, problemas de não aceitação do diagnóstico ou muitas vezes outras coisas por trás disso, que não apenas tomar o medicamento ou não, às vezes questões sobre sexualidade*” (ProfissionalSAE10).

A subcategoria “Entendendo as contradições que envolvem o engajamento ao regime terapêutico” revela duas realidades contraditórias que coexistem. O regime terapêutico proporciona benefícios, principalmente quando atinge uma carga viral indetectável, os medicamentos atuais têm menos efeitos colaterais e há disponibilidade da profilaxia pós-exposição. Contudo, o abandono e o baixo engajamento são uma realidade. A negação da própria condição e postura frente à vida estão entre as causas do abandono e requerem esforços profissionais adicionais para reverter a situação. 

“*Desde o momento que eu descobri, foi tomar os medicamentos, fazer os exames, foi tranquilo, como se não existisse*” (PessoaVHA9).

“*O paciente que desiste do tratamento, será que ele desiste só do tratamento? Como é que é a postura dele frente à vida? As outras coisas, os outros desafios que ele tem? Então, às vezes desistir do tratamento também envolve outros sintomas que possa ter* (...) *uma ansiedade, uma depressão, outras desistências* (...) *que a desistência da medicação está representando também ali, a desistência às vezes da própria vida*” (ProfissionalSAE7).

Pessoas vivendo com HIV/aids enfrentam estigmatização social além do medo, mas também a força da superação, características presentes em duas subcategorias complementares e que se contrapõem simultaneamente. A subcategoria “Desvelando o tabu, preconceito, discriminação e estigma”, retrata o estigma social presente na família, na autopercepção, na relação com e entre os profissionais de saúde e na sociedade.

O preconceito pode ser manifestado em gestos sutis como o modo de olhar comprometendo a ética do cuidado e distanciando os profissionais de sua função essencial. O preconceito em relação ao HIV/aids dificulta a comparação desta com outras condições crônicas. O medo do preconceito associado à vergonha de ser discriminado faz com que as pessoas que vivem com HIV/aids depositem expectativas na sociedade quanto a um acolhimento e reconhecimento que, muitas vezes, elas ainda não dedicam a si próprias. A falta de apoio familiar e da comunidade pode resultar no abandono ou expulsão de casa pela soropositividade e pluralidades de gênero e sexualidade.

“*Na verdade, eles também eram essas pessoas que julgavam, que apontavam que é uma doença* (...) *ainda tem esse pensamento que era uma doença das prostitutas, da promiscuidade, dos gays. Ainda tem muito essa visão de que fez coisa errada que contraiu*” (ProfissionalSAE7).

“*E tem muitas famílias também, quando sabem do* (...) *que o paciente é soropositivo, eles simplesmente abandonam, não querem mais nem saber*” (ProfissionalAPS20).

“...*se tu vais em outro lugar que sabem que é soropositivo, já é* (...)*. Tem uns que já nem conversam, têm medo de dar uma injeção, de pegar na gente, essas coisas* (...)” (PessoaVHA7).

A subcategoria “Vivenciando o medo e a superação profissional frente ao HIV/aids” desvela o medo de adquirir o vírus, ainda que utilize as precauções e a pessoa avise que a carga viral está indetectável. Os profissionais que vivem com HIV também sentem culpa e dificuldade em aceitar que adquiriram o HIV porque conhecem as formas de transmissão. Eles temem transmitir a doença para outras pessoas e buscam se afastar da assistência direta. Apesar disso, há esperança de transformação do estigma reproduzido socialmente.

“*Alguns pacientes até que trabalham no* [nome do local]*. E, muitos até assim querendo deixar a profissão: ‘Não vou mais poder trabalhar com isso’, o medo de passar para outra pessoa*” (ProfissionalSAE7).

“...*mas a gente vai mudando isso ao longo do tempo, em saber que é uma doença totalmente tratável, que pode ter um prognóstico muito bom se seguir o tratamento*” (ProfissionalAPS13).

A última subcategoria “Utilizando sistemas de informação e alguns indicadores”, retrata o desconhecimento dos profissionais da APS acerca de dados do seu território. O SAE monitora o número de testes rápidos realizados, atendimentos e o controle dos exames laboratoriais, de modo individual em cada prontuário e nos sistemas de informação (Sistema de Controle de Exames Laboratoriais de CD4+/CD8+ e Carga Viral do HIV - Siscel, Sistema de Controle Logístico de Medicamentos - SICLOM, e Sistema de Monitoramento Clínico - SIMC). Porém, o controle dos ARV dispensados e das pessoas com HIV/aids que não compareceram para retirar a medicação é manual. Os relatórios dos sistemas de informação são insuficientes para o adequado monitoramento das pessoas e das metas para o enfrentamento da epidemia de HIV/aids. 

“*Nós fazemos o índice de abandono, mas não temos esses dados agora porque começamos a fazer e acabamos abandonando, esquecíamos de anotar. Deveria ser um item que saísse automaticamente no sistema*” (ProfissionalSAE10).

**Figura 1:**
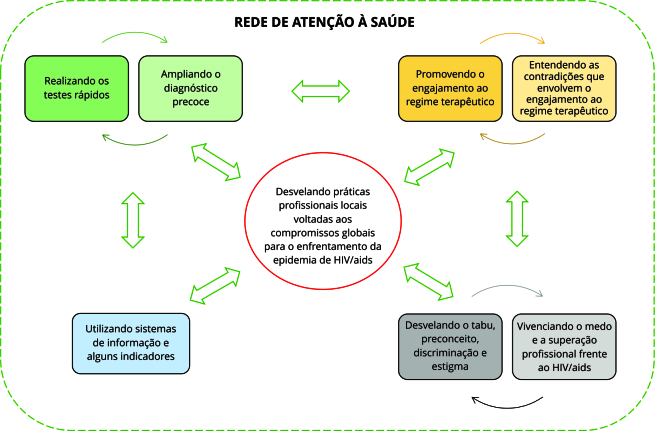
Diagrama das práticas profissionais que buscam atender os compromissos assumidos para o controle a epidemia de aids.

A inter-relação entre as subcategorias deu origem à categoria central “Desvelando práticas profissionais locais voltadas aos compromissos globais para o enfrentamento da epidemia de HIV/aids” (Figura 1). Há aproximações entre as subcategorias que permite visualizar a sua complementaridade para: a realização dos testes rápidos e o diagnóstico precoce; a promoção do engajamento ao regime terapêutico e às contradições que permeiam este cuidado; e o estigma da pessoa que vive com HIV/aids em sua autopercepção, na sociedade e entre os profissionais de saúde. Em meio a esses elementos, ainda há limitações para o adequado monitoramento do progresso das metas globais no contexto local. 

## Discussão

Metas globais com indicadores quantitativos, ainda que mobilizem e estimulem o desenvolvimento de ações voltadas à superação da epidemia de HIV/aids, podem ser consideradas insuficientes para o enfrentamento da problemática, sobretudo na realidade local. A complexidade do ser humano em meio ao mundo fenomênico requer compreender a coexistência de contrariedades na realidade plural e multidimensional, em que a ordem, a desordem, a interação e a organização integram um movimento contínuo, permitindo a compreensão de que jamais será possível escapar da incerteza e atingir um saber total ^19^. 

A ampla implementação de testes para garantir o diagnóstico precoce e início imediato dos ARV, o engajamento do paciente ao tratamento e o alcance da supressão viral resultam em melhores desfechos clínicos ^20^. A disponibilização de testes rápidos na APS a consolida como porta de entrada estratégica para o diagnóstico do HIV, além de estimular a descentralização das ações de cuidado. Tal ampliação não deslegitima o papel dos SAE, cuja atuação permanece essencial. Assim, sugere-se a adoção de um modelo de cuidado compartilhado entre a APS e o SAE, em que o SAE assume a função de apoio matricial, orientando tecnicamente as equipes da APS e fortalecendo a integralidade da atenção ^21^. 

A realização de testes rápidos, aconselhamento e comunicação do diagnóstico pelos enfermeiros destacou-se neste trabalho, conferindo agilidade no acesso, detecção precoce oportuna, e favorecendo a realização da testagem em campanhas e eventos comunitários. Esses achados estão em consonância com o estudo que aponta o enfermeiro como o ator estratégico na realização de testes rápidos para HIV e outras IST, consolidando-se como referência técnica e operacional na APS ^22^.

A realização de testes rápidos na APS requer acolhimento pautado por empatia, vínculo, confiança e sigilo, fundamentais para a aceitação do diagnóstico e o engajamento ao tratamento ^22^. A detecção precoce do HIV é um dos principais objetivos das políticas de enfrentamento da epidemia, e a descentralização por meio dos testes rápidos ampliou o acesso, ainda que limitado em muitos municípios. No entanto, questões relacionadas ao sigilo e à comunicação do diagnóstico podem gerar tensões entre usuários e profissionais, e afetar o vínculo no cuidado ^23^.

A descentralização do diagnóstico e do acompanhamento das pessoas vivendo com HIV/aids para a atenção básica constitui um fenômeno complexo e multifacetado, que gera tensões e potencialidades ^22^. O diagnóstico precoce enfrenta diversos obstáculos como a insuficiência de profissionais ou equipes incompletas, a rotatividade de profissionais, o aumento da carga de trabalho, os questionamentos quanto à confiabilidade dos testes, a ausência de capacitações específicas e a inexperiência quanto ao aconselhamento e à comunicação do diagnóstico ^21^ ^,^ ^24^. Soma-se a isso o apoio matricial incipiente e o risco de desabastecimento de materiais e insumos, comprometendo a continuidade das ações nos serviços de saúde ^21^. Por outro lado, há aspectos favoráveis como o conhecimento e a sensibilidade acerca dos dados epidemiológicos, o trabalho de acordo com os princípios do Sistema Único de Saúde (SUS) e saúde da família, a capacitação, o apoio matricial e a participação da comunidade ^24^. 

Além das metas para 2030, um novo plano com metas e compromissos foi estabelecido para 2025, em busca de reduzir as iniquidades e ampliar a atenção para as pessoas que vivem com HIV e comunidades em risco. Dentre as novas metas, busca-se atingir 95% das mulheres que acessam os serviços de saúde sexual e reprodutiva, e 95% de cobertura de serviços para eliminar a transmissão vertical ^3^. Assim, além da linha de cuidado durante a gestação, é preciso oferecer oportunidades de cuidado para a mulher em todo o ciclo de vida ^25^. Para a prevenção da transmissão vertical do HIV, a APS desempenha papel essencial na cobertura universal de saúde. A oferta de testes rápidos para todas as gestantes e parceiros na APS e a testagem no momento do parto, seguida do tratamento adequado, constituem uma estratégia eficaz ^26^.

O acesso aos ARV é estratégico para alcançar as metas 95-95-95. A oferta gratuita desses medicamentos, como ocorre no Brasil e em grande parte dos países da Europa Ocidental, amplia o acesso e o engajamento ao tratamento, contribuindo para melhores desfechos em saúde ^27^. Embora o município estudado ainda não adote a prescrição de ARV por enfermeiros na APS, esta prática, onde regulamentada, pode reduzir a carga viral e a transmissão do HIV ^28^. Os esquemas atuais, com menor número de doses e menos efeitos adversos, favorecem o engajamento ao tratamento ^8^.

O engajamento ao tratamento do HIV é influenciado por fatores territoriais, como o acesso aos serviços e o receio de exposição ao realizar o tratamento em locais próximos à residência, o que pode gerar medo de estigmatização. O território deve ser compreendido não apenas como espaço físico, mas também em suas dimensões sociais e simbólicas ^12^. O estigma ainda representa um obstáculo, especialmente quando há risco de quebra de sigilo na comunidade.

Modelos diferenciados de prestação de serviços, centrados no paciente e voltados à aceleração dos resultados desejados no tratamento do HIV, priorizam a eficiência sem comprometer a qualidade do cuidado. Para pessoas em condições clínicas estáveis, são ofertadas modalidades como: a dispensação para vários meses; o atendimento rápido; fora do horário comercial; a entrega domiciliar; o modelo escolar; o dia dedicado ao tratamento pediátrico; o apoio ao adolescente; e a clínica para homens ^29^.

Evidências concretas sobre intervenções eficazes para o engajamento à terapia ARV citam ações educativas, uso de lembretes para medicação e apoio de pessoas capacitadas. Os achados destacam a importância de estratégias voltadas tanto aos pacientes quanto aos apoiadores, além da necessidade de qualificar o sistema de saúde e desenvolver intervenções específicas para populações-chave ^30^.

O medo do diagnóstico positivo, suas implicações pessoais e os aspectos culturais em diferentes níveis - individual, interpessoal, comunitário e institucional - contribuem para o estigma e dificultam a busca pelo diagnóstico e o engajamento ao regime terapêutico. O preconceito internalizado e o temor do julgamento social afetam negativamente o diagnóstico precoce, o bem-estar psicológico e a evolução clínica. Esses fatores também impactam a atuação dos profissionais de saúde, comprometendo a oferta de um cuidado adequado às necessidades dos usuários ^22^, e retrata os dilemas e desafios enfrentados no cotidiano dos serviços.

Pessoas que vivem com HIV/aids enfrentam o estigma associado ao vírus/doença, o que gera inseguranças e sofrimento psíquico, afetando diversas dimensões da vida, especialmente os relacionamentos interpessoais e o estilo de vida. Embora o tratamento tenha ampliado a expectativa de vida e contribuído para a desconstrução da imagem da aids como uma “doença da morte”, o estigma permanece como uma barreira significativa ao cuidado. Para enfrentar adequadamente os fatores que impactam o viver com HIV/aids, como a saúde mental e a qualidade de vida, torna-se imprescindível uma assistência integral à saúde, sustentada por uma abordagem interdisciplinar contínua e voltada aos determinantes biopsicossociais ^31^.

Pessoas que vivem com HIV/aids continuam a enfrentar elevados níveis de estigma, o que pode dificultar o acesso aos serviços de saúde devido ao medo de rejeição, além de impactar negativamente sua saúde mental ^32^. O estigma e a discriminação relacionados ao HIV/aids são reconhecidos inclusive entre profissionais da saúde como presentes no próprio ambiente de trabalho ^28^. O estigma relacionado ao HIV resulta na diminuição do acesso ou atraso no recebimento dos cuidados de saúde, ou ainda à discriminação pela equipe de saúde ^32^. Embora o enfrentamento da infecção tenha evoluído ao longo do tempo, persistem resquícios de práticas anteriores, como a rotulação por “grupos de risco”, mantendo-se, assim, a estigmatização na sociedade contemporânea ^28^.

O HIV é uma condição crônica que pode ser controlada devido aos avanços no tratamento ARV. Apesar disso, as pessoas vivendo com HIV/aids enfrentam desafios psicossociais, que resultam em uma pior qualidade de vida, bem como sintomatologia em saúde mental ^33^. O alto nível de estigma relacionado ao HIV interfere na saúde mental das pessoas que vivem com HIV/aids e está associado à maior prevalência de ansiedade e depressão. A perda de amizades e a difamação são preocupações decorrentes da soropositividade ^34^. O estigma faz com que as pessoas tentem esconder seu estado de infecção pelo HIV, o que resulta em início tardio ou interrupção do tratamento ^32^.

Intervenções para a redução do estigma com a criação de ambientes não discriminatórios, e o fortalecimento dos sistemas de apoio e conexão social podem beneficiar a saúde mental das pessoas vivendo com HIV/aids e melhorar a qualidade de vida ^28^. A resposta à epidemia de HIV/aids e à mortalidade a ela associada deve ser integrada às agendas de desenvolvimento territorial, com base em uma abordagem intersetorial e equitativa. Tal integração reconhece que o enfrentamento da epidemia ultrapassa os limites do setor de saúde, demandando o engajamento conjunto de diferentes esferas da sociedade ^35^. 

As metas ambiciosas propostas para o enfrentamento da epidemia de HIV/aids, longe de configurarem um ideal utópico, contribuem para a manutenção contínua do tema nas agendas governamentais. Nesse contexto, destaca-se a importância do engajamento permanente de atores políticos e profissionais de saúde na formulação e implementação de políticas públicas, bem como na promoção da saúde, prevenção de infecções, tratamento e reabilitação. Trata-se de um esforço orientado não apenas à erradicação da aids, mas à ampliação da cidadania, da dignidade e da qualidade de vida das pessoas que vivem com HIV/aids ^2^. 

O enfrentamento da epidemia de HIV/aids exige ações que vão além do setor saúde. A intersetorialidade é essencial para articular políticas públicas que considerem os determinantes sociais da saúde, como educação, habitação e justiça. A equidade, por sua vez, demanda respostas proporcionais às desigualdades vividas por populações historicamente estigmatizadas. Os achados deste trabalho evidenciam boas práticas, mas também os limites estruturais e simbólicos da resposta local, destacando a necessidade de políticas consistentes, o fortalecimento da APS e o enfrentamento do estigma. 

Este trabalho limitou-se à investigação de um único município considerado prioritário para a erradicação da aids, cuja organização do cuidado é centralizada, o que restringe a possibilidade de generalizações. Além disso, adotou-se o recrutamento inicial intencional de participantes, o que impossibilita a generalização de resultados, mas aprofunda as experiências dos sujeitos. Foi possível aprofundar a compreensão do contexto local, apresentando um modelo interpretativo das principais estratégias que vêm sendo utilizadas e dos desafios. 

Ao integrar vozes e práticas locais com os compromissos internacionais, o estudo contribui para o aprimoramento das políticas públicas e para a ampliação do debate sobre equidade, estigma e gestão do cuidado, na medida em que desvela os motivos que interferem nas práticas profissionais para o alcance das metas globais.

Destaca-se o papel de referência do enfermeiro nas práticas de saúde em HIV/aids, especialmente na APS, ressaltando-se sua atuação na gestão e no cuidado direto à pessoa que vive com HIV/aids, na articulação com o SAE e no reconhecimento da rede social destas pessoas como elemento essencial para a superação da discriminação, sobretudo nas relações familiares.

## Considerações finais

O estudo revelou que as práticas profissionais locais voltadas aos compromissos globais para o enfrentamento da epidemia de HIV/aids respondem à questão de pesquisa com base em sete categorias. Esse fenômeno caracteriza-se pelo diagnóstico precoce - principalmente valendo-se da realização de testes rápidos -, a promoção do engajamento das pessoas que vivem com HIV/aids ao regime terapêutico e o acompanhamento destas pessoas por meio do prontuário, indicadores e sistemas de informação. Esse contexto é permeado por contradições e barreiras, como o tabu, o preconceito, a discriminação, o estigma e o medo, mas também pela superação frente ao HIV/aids. Diante disso, as práticas profissionais se configuram como estratégias que orientam o cuidado e intervêm no engajamento ao regime terapêutico. Ainda, a estigmatização social é evidente e denunciada, embora as formas de enfrentamento sejam incipientes.

Para atingir a meta de 95% de pessoas que vivem com HIV/aids diagnosticadas, é necessário ampliar o diagnóstico precoce por meio do fortalecimento do acesso em diferentes serviços de saúde, com acolhimento, aconselhamento, testagem rápida e/ou solicitação de sorologia para HIV, especialmente para gestantes e parceiros, a fim de prevenir a transmissão vertical, além da realização de testagens durante campanhas e eventos, com ênfase em medidas de prevenção e promoção da saúde. Destaca-se o protagonismo do enfermeiro na testagem e no vínculo terapêutico.

Para alcançar a meta de 95% das pessoas diagnosticadas em tratamento antirretroviral, é fundamental a articulação da rede de atenção, com início imediato da terapia e realização de busca ativa nos casos de não engajamento ou abandono. Para atingir os 95% de pessoas em tratamento com carga viral indetectável, torna-se imprescindível o monitoramento do engajamento ao regime terapêutico, exames laboratoriais, garantia de agendamentos e contato telefônico contínuo. É preciso avançar na informatização integrada e na qualificação dos sistemas de informação, ainda frágeis no monitoramento dos indicadores.

As práticas preventivas e clínicas em HIV/aids foram as mais mencionadas pelas equipes de saúde. Entretanto, persistem contradições no engajamento ao regime terapêutico, associadas à estigmatização e à discriminação às pessoas que vivem com HIV/aids - por elas próprias, por seus familiares, por profissionais de saúde e no convívio social. A superação do estigma exige investimentos em educação permanente, formação ética e articulação comunitária, para fortalecer o acolhimento e reduzir as barreiras subjetivas no acesso à saúde. 

Por fim, o enfrentamento da epidemia de aids requer a articulação e a soma de estratégias articuladas, integradas à realidade local e conectadas com o contexto global, além de ações intersetoriais e equitativas voltadas à superação do estigma social e à ampliação do alcance às pessoas que vivem com HIV/aids, promovendo sua saúde e bem-estar.

## Data Availability

As fontes de informação utilizadas no estudo estão indicadas no corpo do artigo.
